# Supplementary material for: Modeling of Astrocyte Networks: Toward Realistic Topology and Dynamics
Source: Front Cell Neurosci. 2021 Mar 5;15:645068. doi: 10.3389/fncel.2021.645068 (PMC7973220; doi:10.3389/fncel.2021.645068)
Supplement: Supplementary file 1 [file Table_1.pdf]

Model parameters

Parameter	Value	Description
c_1	0.185	ER/cytosolic volume ratio
v_1	6.0 s^{-1}	Maximum Ca^{2+} channel flux
d_1	0.13 μM	Dissociation constant for IP_3
d_5	0.082 μM	Ca^{2+} activation constant
v_2	0.11 s^{-1}	Ca^{2+} leak constant
v_3	2.2 $\mu M/s$	Maximum Ca^{2+} uptake
k_1	1.0 s^{-1}	Rate constant of Ca^{2+} extrusion
v_5	0.01 $\mu M/s$	Rate of Ca^{2+} leak across plasma membrane
v_6	0.035 $\mu M/s$	Maximal rate of activation dependent Ca^{2+} influx
k_2	1.0 μM	Half-saturation constant for agonist-dependent Ca^{2+} entry
k_3	0.1 μM	Activation constant for Ca^{2+}-pump
v_4	0.25 $\mu M/s$	Maximal rate of IP_3 production
k_4	1.1 μM	Dissociation constant for Ca^{2+} stimulation of IP_3 production
v_g	0.062 $\mu M/s$	Rate of IP_3 production through glutamate
k_g	0.78 $\mu M/s$	Dissociation constant for glutamate stimulation of IP_3 production
δ	5.0	rate of Ca^{2+} equilibration in ER
α	0.8	
τ_r	7.143 s	Rate constant for loss of IP_3
$IP3_{inf}$	0.16 μM	Steady-state IP_3
d_2	1.049 μM	Dissociation constant for Ca^{2+} inhibition
d_3	0.943 μM	Receptor dissociation constant for IP_3
a_2	0.14 $\mu M s^{-1}$	Ca^{2+} inhibition constant
$[Glu]_{amb}$	0.00 μM	ambient glutamate concentration
τ_{glu}	0.1 $\mu M/s$	rate constant for synaptic glutamate uptake
D_{IP3}	10 $\mu m^2/s$	diffusion coefficient for cytoplasmic IP_3
D_{Ca}	10 $\mu m^2/s$	diffusion coefficient for cytoplasmic Ca^{2+}
D_{Glu}	0.02 $\mu m^2/s$	diffusion coefficient for extracellular glutamate
δ_x	0.275 (single-cell); 0.55 (network)	spatial scale
p_{syn}	0.005–0.01 Hz	rate of Poisson process, for glutamate release
A	27 μM	instantaneous rise in glutamate release rate
$r_{\min}$	0.085	dimensionless & minimal value of the AVF

Underlined entries are new or different from (Ullah et al, 2006). The few values that are different from (Ullah et al, 2006) were adjusted in order to provide the reasonable dynamics with the introduced treatment of $[Ca^{2+}]_{ER}$ as a variable in our model and the spatially extended layout. We had to adjust Ca^{2+}-dependent PLCδ IP_3 production rate, as well as Ca^{2+} extrusion and plasma membrane leakage to correct for areas with high SVR. New parameters dealt with quantal glutamate release as sensed by perisynaptic astrocyte processes. Here we had to choose parameters such that to obtain biophysically plausible model dynamics.

Diffusion coefficient for Ca^{2+} is taken as a lower-bound estimate in Allbritton et al (1992). Slow diffusion coefficient of IP_3 is based on (Dickinson et al, 2016). We also added new parameters, specifically, A, τ_{Glu}, and D_{Glu}. The latter was chosen as a small value to describe only minimal spillover from a release site and buffering by binding to transporters. The pair of parameters describing instantaneous glutamate release rate and slower decay could be varied, because it is hard to assess the actual transmitter concentration and decay time as sensed by astrocyte leaflets. Extracellular glutamate transients occurring due do quantal synaptic release as estimated by fluorescent glutamate sensor have decay timescale in close to 100 ms (Jensen et al, 2019), and this value was used for the simulations shown below. This led to local glutamate transients peaking at 1.2 μM and decaying

within 200 ms. We note that qualitatively similar Ca^{2+} signaling dynamics could be obtained with a shorter τ_{Glu} value, compensated by higher release rate A.

Numerical integration of the model differential equations is done in an explicit scheme (4th order Runge−Kutta method adopted for stochastic differential equations with a fixed timestep $dt = 0.002\,\mathrm{s}$) implemented in AGEOM–CUDA software (Postnov et al, 2012). Spatial grid step was $\delta x = 0.275\,\mu\mathrm{m}$/pixel for single-cell templates and $\delta x = 0.55\,\mu\mathrm{m}$/pixel for network templates (to speed-up simulations). For reproducibilty, a reference implementation of spatial template generation and model simulation is available at `https://zenodo.org/record/4552726#.YDAz1nUzZQ8` in form of Jupyter notebooks, Python and C code.

References

Allbritton NL, Meyer T, Stryer L (1992) Range of messenger action of calcium ion and inositol 1,4,5-trisphosphate. Science 258(5089):1812–5, DOI 10.1126/science.1465619

Dickinson GD, Ellefsen KL, Dawson SP, Pearson JE, Parker I (2016) Hindered cytoplasmic diffusion of inositol trisphosphate restricts its cellular range of action. Science Signaling 9:ra108, DOI 10.1126/scisignal.aag1625

Jensen TP, Zheng K, Cole N, Marvin JS, Looger LL, Rusakov DA (2019) Multiplex imaging relates quantal glutamate release to presynaptic ca. Nat Commun 10(1):1414, DOI 10.1038/s41467-019-09216-8

Postnov DE, Postnov DD, Zhirin R (2012) The "AGEOM_CUDA" software for simulation of oscillatory and wave processes in two-dimensional media of arbitrary geometry on the basis of high-speed parallel computing on graphics processing unit technology CUDA. RF registration certificate #2012610085 from 10.01.2012. (in russian)

Ullah G, Jung P, Cornell-Bell A (2006) Anti-phase calcium oscillations in astrocytes via inositol (1, 4, 5)-trisphosphate regeneration. Cell Calcium 39(3):197 – 208, DOI http://dx.doi.org/10.1016/j.ceca.2005.10.009, URL `http://www.sciencedirect.com/science/article/pii/S0143416005002083`
